# An Ab Initio Investigation of the 4,4′-Methlylene Diphenyl Diamine (4,4′-MDA) Formation from the Reaction of Aniline with Formaldehyde

**DOI:** 10.3390/polym11030398

**Published:** 2019-03-01

**Authors:** R. Zsanett Boros, László Farkas, Károly Nehéz, Béla Viskolcz, Milán Szőri

**Affiliations:** 1 Wanhua-BorsodChem Zrt, Bolyai tér 1., H-3700 Kazincbarcika, Hungary; renata.boros@borsodchem.eu (R.Z.B.); laszlo.farkas@borsodchem.eu (L.F.); 2 Institute of Chemistry, University of Miskolc, Miskolc-Egyetemváros A/2, H-3515 Miskolc, Hungary; 3 Department of Information Engineering, University of Miskolc, Miskolc-Egyetemváros Informatics Building, H-3515 Miskolc, Hungary; aitnehez@uni-miskolc.hu

**Keywords:** bis(4-aminophenyl)methane, MDA, PABA, aniline, water, reaction mechanism, ab initio, G3MP2B3, transition state, p*K*_a_, standard enthalpy of formation

## Abstract

The most commonly applied industrial synthesis of 4,4′-methylene diphenyl diamine (4,4′-MDA), an important polyurethane intermediate, is the reaction of aniline and formaldehyde. Molecular understanding of the 4,4′-MDA formation can provide strategy to prevent from side reactions. In this work, a molecular mechanism consisted of eight consecutive, elementary reaction steps from anilines and formaldehyde to the formation of 4,4′-MDA in acidic media is proposed using accurate G3MP2B3 composite quantum chemical method. Then G3MP2B3-SMD results in aqueous and aniline solutions were compared to the gas phase mechanism. Based on the gas phase calculations standard enthalpy of formation, entropy and heat capacity values were evaluated using G3MP2B3 results for intermediates The proposed mechanism was critically evaluated and important side reactions are considered: the competition of formation of protonated p-aminobenzylaniline (PABAH^+^), protonated aminal (AMH^+^) and o-aminobenzylaniline (OABAH^+^). Competing reactions of the 4,4′-MDA formation is also thermodynamically analyzed such as the formation of 2,4-MDAH^+^, 3,4-MDAH^+^. AMH^+^ can be formed through loose transition state, but it becomes kinetic dead-end, while formation of significant amount of 2,4-MDA is plausible through low-lying transition state. The acid strength of the key intermediates such as N-methylenebenzeneanilium, PABAH^+^, 4-methylidenecyclohexa-2,5-diene-1-iminium, and AMH^+^ was estimated by relative p*K*_a_ calculation.

## 1. Introduction

Polyurethane industry requires large amount of methylene diphenyl diisocyanate (MDI) as raw material and global market of MDI reached 6 Mt in 2016 [1]. The MDI production is mostly based on methylene diphenyl diamine (methylenedianiline, MDA). MDA can be also applied as ingredient of epoxy resins, intermediate for pigments, organic dyes, coatings, plastic fibers, and insulation materials [2] making it an important intermediate for chemical industry.

**Figure 1:**
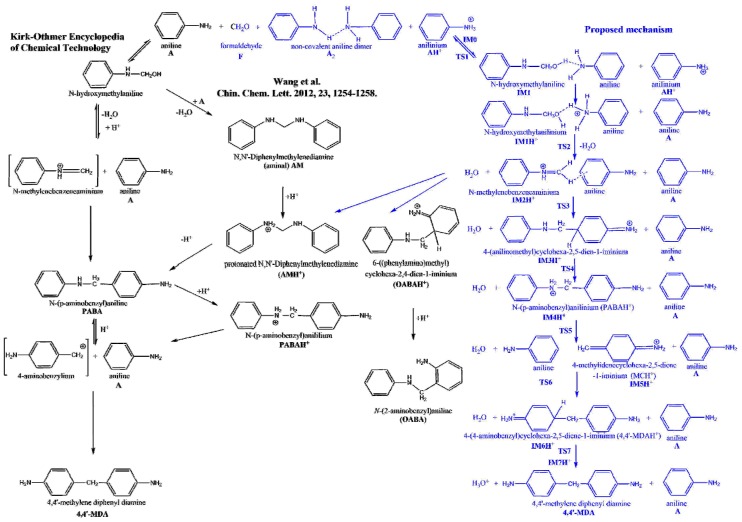
Overall reaction mechanism for methylene diphenyl diamine (methylenedianiline, MDA) synthesis according Kirk–Othmer Encyclopedia of Chemical Technology [12] as well as Wang [13] compared with our proposed mechanism based on G3MP2B3 computation.

The most commonly applied industrial MDA synthesis is the reaction of aniline with formalin in the presence of hydrochloric acid under mild (60–110 °C) reaction conditions [3]. To avoid the use of corrosive hydrochloric acid and the formation of large amount of salt solution as waste, several attempts [4,5,6,7,8,9,10,11] had been made to replace the current technology with catalytic process using solid acids, zeolites, delaminated materials, ionic liquids, or ion exchange resins as catalysts. However, neither of the catalytic MDA productions have gone beyond the laboratory stage. Although, proposed reaction mechanism for current MDA synthesis is presented in Ref. [12] (see left side of Figure 1), it has not been clarified entirely. According to this mechanism, aniline (A) is reacted with formaldehyde (F) producing N-hydroxymethyl aniline as the initial reaction step. In acidic medium this product loses water rapidly to form N-methylidene anilinium which reacts with aniline to form N-(p-aminobenzyl)aniline (PABA). PABA is then decomposed to 4-aminobenzylium and aniline. In the last step of this rearrangement 4,4′-methylene diphenyl diamine (4,4′-MDA) is formed as final product. Beside missing elementary steps, this early mechanism also cannot clarify the detected side products of MDA.

More recently Wang et al. [13] suggested an alternative mechanism in which the reaction of N-hydroxymethylamine and aniline (A) gives N,N’-diphenylmethylenediamine first (so-called aminal noted as AM in Figure 1) before the formation of PABA. Due to the protonation of one of the secondary amine group in AM (AMH^+^), the C-N bond is activated for the rearrangement making PABA. According to Wang, same activation is supposed to happen to the other secondary amine group to form MDA: PABA is protonated (PABAH^+^) which then initiates aniline rearrangement. Wang et al. found some evidences to support the latter mechanism by isolation and identification of aminal using the combination of isotope labeling and HPLC-MS [13]. Albeit other intermediates has not been characterized at all. In the same study by-products including oligomers of MDA (e.g., 3- and 4-ring MDA) were also suggested. As a continuation of this work stabilities, potential protonation sites and structural characterization of these MDA oligomers were determined using ion mobility-mass spectrometry (IM-MS) and tandem mass spectrometry (MS/MS) techniques [14]. This resulted in two partially overlapping, but still gappy mechanism for MDA production as seen from the left side and the center of Figure 1. Furthermore, initial step of both mechanisms requires close contact of aniline and aqueous formaldehyde. Although aniline and water have similar density [15], but they are only slightly soluble in each other and aniline has large viscosity (3.770 cP) due to strong hydrogen bond between amine groups, [16] therefore, solvent effect can be crucial in the MDA production for these solvents.

Although, the MDA synthesis is essential to MDI production, the thermochemical properties of the participated species are also poorly characterized [17]. To the best of our knowledge, only the standard reaction enthalpy of formation for MDA, PABA, and AM is estimated using Benson group additivity method [18]. Only the heat of formation of 4,4′-MDA had been reported very recently using G3MP2B3 quantum chemistry protocol and group additivity increments for OCN and NHCOCl groups has been recommended [19] based on protocols mentioned in Ref. [20,21].

The aim of this study is to establish an ab initio-based molecular mechanism consisting of consecutive elementary reaction steps from anilines and formaldehyde to the formation of 4,4′-MDA. These elementary reactions can include short living species with low steady state concentrations which makes them inaccessible for most of the current detection techniques. Furthermore, we characterized thermodynamically the detailed reaction mechanism of the current industrial MDA production using quantum chemical calculations. The effects of the surrounding media as well as the protonation state of the intermediates is also characterized. Few important competing reaction channels are also included to provide theoretically established evidence for by-products.

## 2. Methods

G3MP2B3 [22] composite method was applied for obtaining thermodynamic properties such as zero-point corrected relative energy (Δ*E*_0_), relative enthalpy (Δ*H*), standard enthalpy of formation (Δ_f,298.15K_*H*(g)), relative molar Gibbs free energy (Δ*G*), standard molar entropy (*S*), and heat capacity (*C_V_*) for the species involved in the reaction mechanism of the formation of MDA from the reaction of aniline molecules with formaldehyde in acidic medium. As part of G3MP2B3 protocol B3LYP [23] functional was applied in combination with the 6-31G(d) basis set for geometry optimizations and frequency calculations, except for solvated TS2 structures (Since B3LYP/6-31G(d) was unable to localize these transition states (TS), it was replaced by BH&HLYP/6-31G(d) level of theory). Normal mode analysis was performed on the optimized structures at the same level of theory to characterize their identities on the potential energy surface (PES). TS structures were also checked by visual inspection of the intramolecular motions corresponding to the imaginary wavenumber using GaussView05 [24] as well as confirmed by intrinsic reaction coordinate (IRC) calculations [25] for mapping out the minimal energy pathways (MEP).

Although accuracy of G3MP2B3 is proven in our previous work for similar reaction system [19], gas phase standard heat of formation values obtained at G3MP2B3 and CBS-QB3 [26] computations were compared for further verification purposes. The gas phase standard heat of formation values at T = 298.15K, Δ_f,298.15K_*H*(g), were achieved using an atomization scheme (AS) [27] and isodesmic reaction (IR). Accurate literature data necessary for AS calculation was collected from Ruscic’s Active Thermochemistry Tables [28].

SMD polarizable continuum model developed by Truhlar and co-workers [29] was used to estimate the effect of the surrounding solvent (water and aniline). The acid dissociation constant (p*K*_a,aq_) in aqueous solution of the intermediates were also derived from G3MP2B3 calculations. The p*K*_a,aq_ values can be calculated using Gibbs free energy of the gas phase species in neutral and cationic form (noted as G(A_g_) and G(AH^+^_g_), respectively) which corrected by the solvation Gibbs free energies (Δ*G*_aq_) of the species involved [30]:

p*K*_a,aq_ = [*G*(A_g_) − *G*(AH^+^_g_) + Δ*G*_aq_(A) − Δ*G*_aq_(AH^+^) + *G*_g_(H^+^) + *G*_aq_(H^+^) + RTln(*V*_m_)]/R*T*ln10, (1)

where gas phase Gibbs free energy for proton *G*_g_(H^+^) comes from Sackur-Tetrode equation (26.3 kJ/mol [31]) and solvation Gibbs free energy for proton, Δ*G*_aq_(H^+^), is −1107.1 kJ/mol [32]. Molar volume (*V*_m_) at reference state is 24.46 dm^3^. Although, such direct p*K*_a_ calculation via thermodynamics cycle usually suffers from larger error, therefore proton exchange scheme was applied to improve the accuracy of a p*K*_a_ calculation (p*K*_a_ of anilinium, AH^+^, used as reference). All quantum chemical calculations were performed by Gaussian09 [33] software package.

## 3. Results and Discussion

### 3.1. Main Reaction Mechanism in Gas Phase

Similar to our previous study [19] gas phase mechanism is used as reference to measure the solvent effect as the presence of solvent can make a significant difference on the reaction energy profile.

**Figure 2:**
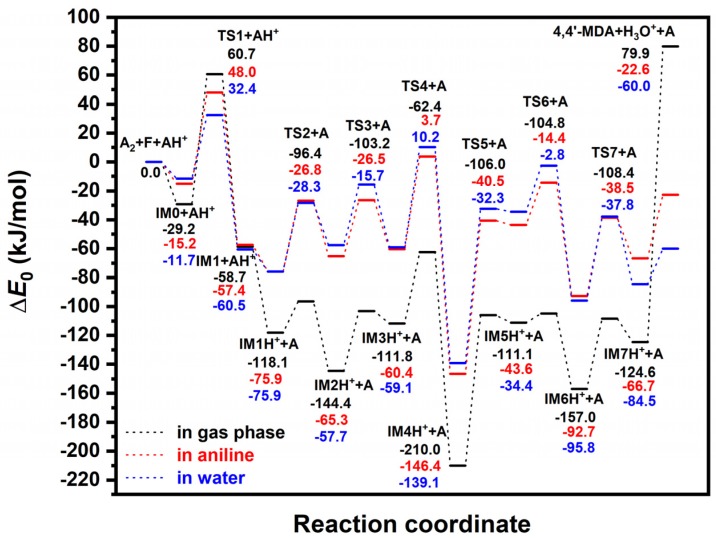
G3MPB3 energy profile (zero-point corrected) for MDA synthesis in gas phase (black), in aniline (red) and in water (blue).

According to the X-ray scattering experiment of aniline [16], closest intermolecular distance between nitrogen atoms in a pair of aniline (A) molecules is 3.31 Å, which is close to the distance (3.159 Å) found in the V-shape non-covalent bonded aniline dimer (A_2_) computed at G3MP2B3 in gas phase. This short distance and orientational preference are due to the strong hydrogen bond between two amine groups explaining the large viscosity of liquid aniline [16], which is in line with our G3MP2B3 results (the formation of the gas phase non-covalent aniline dimer is −19.5 kJ/mol in term of zero-point corrected energy). Therefore, in our calculations, A_2_ structure had been selected as initial structure (see right side of the Figure 1). The energy reference used in Figure 2 (also given in Table 1) corresponds to the sum of non-covalent A_2_ dimer, formaldehyde (F) and protonated aniline (AH^+^). The latter species supposes to mimic the acidic environment used by chemical industry.

**Figure 3:**
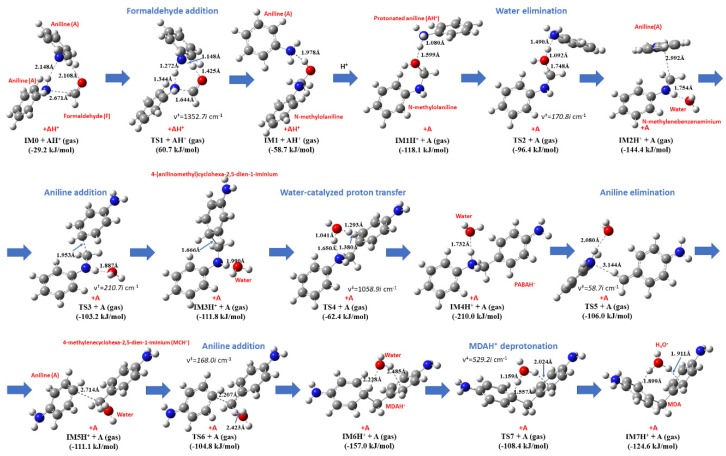
Transition state structures (obtained at B3LYP/6-31G(d) level of theory) for MDA synthesis in gas phase. The G3MP2B3 relative energies are also given.

The protonated aniline dimer (A_2_) approaches the formaldehyde (F) to form the pre-reactive complex (IM0, see Figure 3), in which formaldehyde is strongly hydrogen bonded to one of the anilines (r_O-H_ = 2.108 Å) and its carbon atom approaches the nitrogen atom of the other amine (r_C-N_ = 2.671 Å). This structure is 29.2 kJ/mol lower in energy compared to the separated formaldehyde, protonated aniline and aniline dimer (reference state). As seen in Figure 3, the transition state for formaldehyde addition to the aniline (TS1) is a six-membered ring structure, where the carbon of F got closer to the nitrogen of the amine by roughly 1 Å (r_C-N_ = 1.644 Å) compared to the IM0. Simultaneously, attacked nitrogen releases a hydrogen which is transferred to the oxygen of formaldehyde through the other aniline amine group. The corresponding energy is 60.7 kJ/mol higher than that of the reactants making this structure as the highest energy TS along the entire reaction mechanism studied here. As next step, the resulted N-hydroxymethylaniline (IM1) forms molecular complex with protonated aniline (AH^+^), noted as IM1H^+^, in which the protonated amine group is in vicinity of the OH group of N-hydroxymethylaniline (r_OH_ = 1.599 Å). This protonated complex has significantly lower in energy (−118.1 kJ/mol) compared to the previous neutral complex (−58.7 kJ/mol).

Transition state of the water elimination (TS2) is structurally similar to the previous IM1H^+^, although the O-H being formed become significantly shorter (r_O-H_ = 1.092 Å), while the C-O bond expanded to 1.748 Å. TS2 is 21.7 kJ/mol higher than IM1H^+^. The product of this exothermic reaction (IM2H^+^) is a trimolecular complex (aniline-water and N-methylenebenzeneaminium) having −144.4 kJ/mol of relative energy. In this structure, the carbon of the aniline at para position is just 2.992 Å far from the methylene group of N-methylenebenzenaminium and the water oxygen is hydrogen bonded to the hydrogen atom of the secondary amine group. The critical C-C distance is 1.953 Å in the transition state structure of the aniline addition (TS3) and water is only slightly rotated around the elongated hydrogen bond (1.887 Å). The activation energy of TS3 is 41.2 kJ/mol, while its relative energy become −103.2 kJ/mol. By linking these two aromatic ring structures, water complex of 4-(anilinomethyl)cyclohexa-2,5-dien-1-iminium is formed (IM3H^+^) in a slightly endothermic reaction (Δ_r_*H* = 29.5 kJ/mol obtained from data in Table 1). Despite the partial loss of the aromatic nature of the aniline, the IM3H^+^ structure is −111.8 kJ/mol lower than the reference energy. In the next step of the proposed mechanism, the water reoriented to initiate the transfer of the positive charge to the amine nitrogen in such a way that the second aromatic ring can also be formed (TS4). This six-centered transition state with the activation energy of 49.4 kJ/mol resulted in the product (noted as IM4H^+^) with the lowest relative energy (Δ*E*_0_(IM4H^+^) = −210.0 kJ/mol) structures in the entire mechanism, the hydrogen bonded complex of the N-(p-aminobenzyl)anilinium (PABAH^+^) and water. Due to the proton of the amine, PABAH^+^ is activated to dissociate to aniline (A) and 4-methylidenecyclohexa-2,5-diene-1-iminium (MCH^+^) noted as IM5H^+^ via C-C bond scission (TS5). The activation energy of this reaction step is 104.0 kJ/mol. To make rearrangement of the released aniline happen, the formed PABAH^+^ should be chemically activated and stabilization of PABAH^+^ should be avoided (e.g., through deprotonation of PABAH^+^). Firstly, we considered the aniline addition occurs at para position to the MCH^+^ to form protonated 4,4′-methylenedianiline (4,4′-MDAH^+^) noted as IM6H^+^. After the formation of the loosely bounded complex of MCH^+^ and aniline (IM5H^+^), these two species can form TS6 structure in which C-C bonds being formed is 2.207 Å. Only small rotational motion of the water molecule contributes to the reaction coordinate (ν^‡^ = 168.0*i* cm^−1^) in this case. The energy level of TS6 is −104.8 kJ/mol compared to the entrance level.

In IM6H^+^, water binds to the MDAH^+^ by hydrogen bond and interaction occurs of its other hydrogen and the aromatic ring. This structure shows similarity for the deprotonation transition state, that is TS7, in which the distance of C-H being broken is significantly elongated (1.557 Å) and the critical distance of H-O bond is short (1.159 Å). Moving along the reaction coordinate the water molecule reoriented again and its one of the alone pairs is now pointed toward the extra proton of IM6H^+^ making the post-reaction complex, IM7H^+^ after a slight reorientation of the H_3_O^+^ cation. This structural change resulted in an energy decrease by 16.2 kJ/mol (Δ*E*_0_(IM7H^+^) = −124.6 kJ/mol). The relative energy of last three transition states is also energetically close to each other (Δ*E*_0_(TS5) = −106.0 kJ/mol, Δ*E*_0_(TS6) = −104.8 kJ/mol, and Δ*E*_0_(TS7) = −108.4 kJ/mol). Finally, the post-reaction complex converted into the final product that is the 4,4′-MDA (Δ*E*_0_(4,4′-MDA) = 79.9 kJ/mol) which has the highest relative energy considering the whole reaction coordinate in gas phase. Basically, this consistent mechanism can be considered as an extension of the mechanism found in Kirk–Othmer Encyclopedia of Chemical Technology [12] by three new intermediate elementary steps (formation of IM3H^+^, IM4H^+^, and IM6H^+^). No experimental evidence found for 4-(aminomethyl)cyclohexa-2,5-dien-1-iminium (part of IM3H^+^ complex) as well as protonated 4,4′-MDA (part of IM6H^+^ complex). One plausible reason for that is their destroyed aromatic nature making them short living and strong acid. Although, amongst these new species, IM4H^+^ differs only in the protonation state from the PABA intermediate suggested in Ref. [12]. On the other hand, the one step formation of aminal (AM) suggested by Wang [13] is unlikely, since the corresponding hypothetical transition state would be crowded around the methylene carbon, therefore reaction of N-methylenebenzeneaminium and aniline is considered as a formation of protonated aminal (AMH^+^) instead (for its detailed discussion see Important side reactions section). Before the solvent effect is discussed the gas phase thermochemistry of the species playing role is evaluated.

### 3.2. Gas Phase Thermodynamic Properties of Reactants, Intermediates, and Products

As shown in Table 2, the calculated standard enthalpies of formation for aniline, 4,4′ MDA and all intermediates are endothermic, while the formation of formaldehyde (−111.5 kJ/mol) and N-hydroxymethylaniline (−71.8 kJ/mol) are exothermic.

Highly accurate standard enthalpy of formation (Δ_f,298.15K_*H*^0^) value is only reported for aniline and formaldehyde in the literature [28,34]. The largest error for their G3MP2B3 computation is 2.3 kJ/mol, which is significantly smaller than those for CBS-QB3, therefore only the G3MP2B3 results are discussed latter. While the G3MP2B3 value for 4,4′-MDA (171.2 kJ/mol) is also consistent with the estimated values based on group additivity rules by Benson [18] and Benson and Stein [36,37,38] (165.6 kJ/mol [11] and 172.0 kJ/mol, respectively), only CBS-QB3 estimates more endothermic enthalpy of formation for 4,4′-MDA. Interestingly, ortho and meta isomers of MDA (2,4-MDA, 2′,4-MDA, and 3,4-MDA) have less endothermic formation than 4,4′-MDA, while among their protonated forms 2,4-MDA and 4,4′-MDA found to be less endothermic. For these species, only modest difference in molar entropy had been found. Furthermore, G3MP2B3 and the group additivity values are also consistent with each other in the case of PABA. To the best of our knowledge, no literature Δ_f,298.15K_*H*^0^ was found for other intermediates presented here (Table 2). The computed standard molar entropy (*S*°(g)) and molar heat capacity (*C*_V_(g)) values are also tabulated in Table 2, and their deviation from accurate literature values [34,35] is less than 8.6 J/molK, while larger deviation is observed from the results obtained form group additivity (22.6 J/molK for 4,4′-MDA) which is probably due to missing correction terms in the group additivity.

### 3.3. Solvent Effect

The presence of solvents (aniline and water) changes significantly energy profile of the reaction as shown in Figure 2, the solvent effect is very similar regardless which solvent is considered. The most dramatic change is the stabilization of the final product (see 4,4′-MDA+H_3_O^+^+A in Table 1) by 102.5 kJ/mol for aniline solution and by 139.9 kJ/mol for aqueous solution which is mainly due to the solvation energy difference between protonated aniline (AH^+^) and hydronium cation (H_3_O^+^). The highest lying barrier in gas phase, that is TS1, is also decreased due to solvation by 12.7 kJ/mol (in aniline) and 28.3 kJ/mol (in water). While its pre-reaction (IM0 + AH^+^) and post-reaction (IM1 + AH^+^) complexes are destabilized slightly (14.0 kJ/mol and 1.3 kJ/mol in aniline, respectively), large destabilization effect can be observed for the intermediates and transition states after the protonation of IM1 intermediates (IMx and TSx, where 2 ≤ x), its magnitude is in the range of 42.2–90.5 kJ/mol for the aniline solution, while it is 40.1–102.1 kJ/mol for the aqueous solution (about 60 kJ/mol in average). The largest increase in relative energy belongs to the transition state of the second aniline addition (TS6) for both solutions, this energy shift was 90.5 kJ/mol and 102.1 kJ/mol for aniline and for aqueous solution, respectively. Similarly, the transition state for first aniline addition (TS3) and its pre-complex (IM2H^+^) are also significantly destabilized by solvation compared to the other TSs and intermediates (in the range of 76.8–87.5 kJ/mol). Structural changes according to solvent effect are shown in the Supplementary Materials (see Figure S1 and Figure S2 in for aqueous and aniline solution, respectively).

As Table 1 shows, relative enthalpies (Δ*H*^0^) show same trend as Δ*E*_0_, Δ*H*^0^ values tend to be larger, but not more than 26.6 kJ/mol. Most of the cases, difference in relative enthalpies (ΔΔ*H*^0^_an__→aq_) by comparing aniline to aqueous phase is less than 9.2 kJ/mol. Larger difference in Δ*H*^0^ values found in those cases, where large solvation effect for Δ*E*_0_ had already been observed, such as TS1, TS3, TS6, IM7H^+^ and 4,4′-MDA (ΔΔ*H*^0^_an__→aq_(TS1) = −15.6 kJ/mol, ΔΔ*H*^0^_an__→aq_(TS3) = 10.7 kJ/mol, ΔΔ*H*^0^_an__→aq_(TS6) = 11.6 kJ/mol, ΔΔ*H*^0^_an__→aq_(IM7H^+^) = −17.8 kJ/mol, and ΔΔ*H*^0^_an__→aq_(4,4′MDA) = −37.3 kJ/mol). Analysis of the relative Gibbs free energies (Δ*G*^0^) in Table 1 show also that solvation Gibbs free energies (Δ*G*_aq_^0^) and Δ*E*_0,aq_ are in linear relationship for both surrounding media.

### 3.4. Proton Dissociation Constants (pK_a_) of Intermediates

The species in proposed mechanism are proton activated, their possible deprotonation can lead to side reactions and appearance of deactivated intermediates in the industrial process. Therefore, the affinity of these species for deprotonation is important and it can be described by site-specific acid dissociation constant (p*K*_a_) [39,40]. Ghalami-Choobar et al. performed calculation for aqueous p*K*_b_ values of aniline and its substituted derivatives with good accuracy [41]. Behjatmanesh-Ardakani [42] and Lu [43] also reported calculated p*K*_a_ for some aniline derivatives. Although, direct p*K*_a_ calculation via thermodynamics cycle usually suffers from larger error than the relative method. In this study, the G3MP2B3-SMD based absolute p*K*_a_ value of anilinium (AH^+^) was found to be 2.86 in aqueous phase using the direct p*K*_a_ estimation which is smaller by 1.74 p*K*_a_ units than the reference value (p*K*_a,aq_ = 4.60 in Ref. [44], so we have used the deprotonation half-reaction of anilinium as reference for the relative p*K*_a_ calculation shown in Table 3.

N-methylenebenzeneanilium, N-(p-aminobenzyl)anilinium and PABAH^+^ have similar p*K*_a_ values which corresponds to weak acid and they can lose their potential to turn into MDA by deprotonation, while acid strength of MCH^+^ is far less therefore it likes to be protonated. Indeed, instead of PABAH^+^_,_ deprotonated PABAH^+^ (PABA) has been detected from aniline-formaldehyde condensation mixture [45].

### 3.5. Important Side Reactions

Alternative aniline addition reactions can also be proposed. One of these side reactions can be the protonated aminal formation (see AMH^+^ in Figure 1), which is essentially an alternative to the formation of IM3H^+^ from aniline to the N-methylenebenzeneaminium (‘the first’ aniline addition). However, the aniline addition occurs through the amine group instead of the aromatic carbon at para position. Interestingly, loose transition state had been found for this reaction in both condensed media, which was also proven by scan of the potential energy surface via the C-N bond stretching of the protonated aminal at B3LYP/6-31G(d) level of theory. The B3LYP curve was also reproduced by BHandHLYP and MP2 methods. In contrast, the formation of ortho adduct (o-aminobenzylaniline, OABAH^+^) undergoes tight submerged transition state (Δ^‡^*E*_0_ = −9.4 kJ/mol in aniline phase, Δ^‡^*E*_0_ = −5.0 kJ/mol in aqueous phase).

As seen from Table 4, aminal (AMH^+^) formation reaction is exothermic, but it has only moderate exergonicity in each media studied in contrast to the competitive reaction step, IM3H^+^ formation. However, the thermochemical and kinetic favor of formation of AMH^+^ over IM3H^+^ is obvious, AMH^+^ is without relevant new exit channel. Similarly, formation of OABAH^+^ is more exothermic than that of IM3H^+^, although it is more endergonic by 32 kJ/mol than in the case of IM3H^+^.

Beside the formation of 4,4′-MDAH^+^, the aniline addition to MCH^+^ (second aniline addition) can also result in the formation of ortho and meta MDAH^+^ isomers (2,4-MDAH^+^, 2′,4-MDAH^+^ and 3,4-MDAH^+^). The thermodynamic properties of these competitive reactions are collected in Table 5.

**Figure 4:**
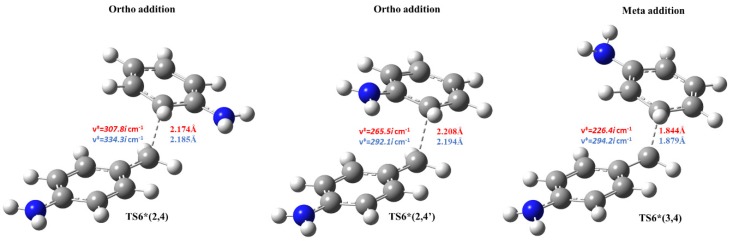
Transition state structures (obtained at B3LYP/6-31G(d) level of theory) for 4,4′-MDA isomer formation (Critical distance are also given for both condensed phases).

From both thermodynamic and kinetic points of view, aniline addition to ortho position (2,4) is at least as preferred as the para position (4,4) due to the low activation energy and energy release. In contrast, the meta addition (3,4) is endothermic with high activation barrier in both condensed phases. More interestingly, one of the ortho addition steps has submerged transition state (−1.9 kJ/mol in aqueous phase) due to the strong ion-dipole interaction manifested in the vicinity (2.2 Å) of amine group of A and benzene ring of MCH^+^ as shown in Figure 4.

**Figure 5:**
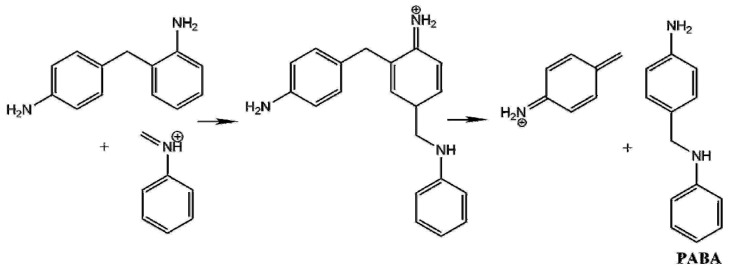
Schematic reaction mechanism of the formation of the 3-ring adduct and its dissociation to N-methylenebenzeneaminium and PABA.

Based on these results, significant amount of 2,4-MDA should be formed as a product of the title reaction which is in clear contradiction with laboratory observations (more than 92 *w*/*w*% of the product is 4,4′-MDA while 7 *w*/*w*% is 2,4′-MDA [46]). This contradiction might be caused by incomplete quantum chemical description of the solvent effect and/or neglecting the role of the counter ion in the mechanism. On the other hand, 33.5 *w*/*w*% of the product is oligomeric and polymeric MDA [46]. Another hypothesis might be that the 2,4′-MDA is more reactive towards further aniline addition making oligomeric structures (e.g., 3-ring) overrepresented in these forms. To bear in mind that the above mentioned experiments are on the several minutes time scale, that makes reactive interferences between the intermediates and products possible (the w% of 4,4′-MDA became saturated at 20% after ca. 40 min in Knjasev experiment at T = 70 °C, where the initial molar ratio of the mixture was A:F:HCl:H_2_O = 4:2:1:13 [45]). An example for such interference is the highly preferred addition of the N-methylenebenzeneaminium (the ‘3-ring’ structure) in the para position which is also supported by the ^2^H- and ^13^C-NMR-based observation by Knjasev [45]. This step can be followed by proton transfer which might make the dissociation of the adduct favorable to MCH^+^ and PABA as it shown in Figure 5.

## 4. Conclusions

Reaction mechanism of the MDA formation from aniline and formaldehyde was determined using G3MP2B3 quantum chemical method in gas phase and in industrially relevant solvents such as aniline and water. The non-covalent aniline dimer approaches formaldehyde to form the prereactive complex from which eight elementary reaction steps resulted in 4,4′-MDA as the final product. Our important findings for the whole reaction mechanism are:

1. The highest lying transition state (60.7 kJ/mol) corresponds to formaldehyde addition to aniline (TS1) leading to N-hydroxymethylaniline formation and its barrier heights significantly decreased by solvation. This step can be the main kinetic bottleneck for 4,4′-MDA production. After exothermic water elimination, aniline addition to N-methylenebenzenaminium took place through either tight transition state (resulted in 4-methylidenecyclohexa-2,5-diene-1-iminium) or loose transition state to form protonated aminal. However, aminal formation is both thermodynamically and kinetically preferred, there is no exit channel belong to it. Afterwards, proton shift can undergo in 4-methylidenecyclohexa-2,5-diene-1-iminium to produce protonated PABA, PABAH^+^, being the global minimum at this reactive potential energy surface, which can be also along with the line with experimental observation of PABA during MDA production. Two steps rearrangement of aniline resulted the protonated MDA isomers (MDAH^+^). It was found that aniline addition in ortho position is competitive with that of in the para position from both kinetic and thermodynamic points of view. The deprotonation of MDAH^+^ is thermodynamically more favorable in water phase.
2. The species in proposed mechanism are proton activated, their possible deprotonation can lead to side reactions and appearance of deactivated intermediates in the industrial process. Therefore, the acid strength of four important intermediates such as N-methylenebenzeneanilium (4.2), PABAH^+^ (6.7), MCH^+^ (11.4), and AMH^+^ (5.1) was estimated using relative p*K*_a_ calculation. Although, most of them found to be weak acid in aqueous solution, but they got more acidic in aniline (basic) environment which can then deactivate the intermediates.
3. Aniline addition-type side reactions had been also investigated and it was found that aminal formation is both thermodynamically and kinetically preferable, but it is a kinetic dead-end. Both 2,4- and 4,4′-MDAH^+^ formation from MCH^+^ and A has low-lying transition state and TS is submerged in the case of 2,4-MDAH^+^ making likely the formation of 2,4-MDAH^+^ beside 4,4-MDAH^+^.
4. Gas phase thermodynamic properties for the reactants, products and intermediates were determined and carefully compared to the literature. Based on our G3MP2B3 and CBS-QB3 calculations, accurate standard enthalpy of formation is recommended for the intermediates.

## Figures and Tables

**Table 1:** G3MP2B3 thermochemical properties calculated in gas phase, in aniline and in water including zero-point corrected relative energies (Δ*E*_0_), relative enthalpies (Δ*H*(T)) and relative Gibbs free energies (Δ*G*(T,P)) at T = 273.15 K, and P = 1 atm.

Species	Δ*E*_0_			Δ*H*(T)			Δ*G*(T,P)		
	Gas	Aniline	Water	Gas	Aniline	Water	Gas	Aniline	Water
**A_2_ + F + AH^+^**	0.0	0.0	0.0	0.0	0.0	0.0	0.0	0.0	0.0
**IM0 + AH^+^**	−29.2	−15.2	−11.7	−30.0	−15.2	−11.7	17.2	26.3	31.3
**TS1 + AH^+^**	60.7	48.0	32.4	52.4	40.2	24.6	121.7	106.3	92.5
**IM1 + AH^+^**	−58.7	−57.4	−60.5	−64.0	−61.9	−65.6	−6.2	−10.1	−8.3
**IM1H^+^ + A**	−118.1	−75.9	−75.9	−123.3	−80.3	−81.0	−68.6	−26.4	−23.8
**TS2 + A**	−96.4	−26.8	−28.3	−121.6	−53.0	−53.8	38.2	106.2	102.3
**IM2H^+^ + A**	−144.4	−65.3	−57.7	−162.4	−83.2	−75.2	−18.5	58.0	64.0
**TS3 + A**	−103.2	−26.4	−15.7	−124.4	−46.3	−35.1	30.9	104.1	114.2
**IM3H^+^ + A**	−111.8	−60.4	−59.1	−132.9	−80.5	−79.6	23.8	70.0	72.7
**TS4 + A**	−62.4	3.7	10.2	−88.3	−20.5	−13.6	81.1	141.0	148.1
**IM4H^+^ + A**	−210.0	−146.4	−139.1	−231.8	−167.5	−160.5	−72.9	−14.6	−6.9
**TS5 + A**	−106.0	−40.5	−32.3	−125.0	−57.7	−49.9	21.0	79.9	87.8
**IM5H^+^ + A**	−111.1	−43.6	−34.4	−127.3	−58.9	−49.3	15.1	72.3	83.0
**TS6 + A**	−104.8	−14.4	−2.8	−123.0	−29.9	−20.7	28.0	108.8	128.2
**IM6H^+^ + A**	−157.0	−92.7	−95.8	−177.3	−110.7	−114.3	−20.0	34.5	33.4
**TS7 + A**	−108.4	−38.5	−37.8	−132.3	−60.5	−60.1	35.7	98.4	101.2
**IM7H^+^ + A**	−124.6	−66.7	−84.5	−147.3	−88.1	−06.3	17.0	69.0	53.1
**4,4′-MDA + H_3_O^+^ + A**	79.9	−22.6	−60.0	60.0	−41.5	−79.3	176.7	67.6	32.8

**Table 2:** Gas phase thermochemical properties for reactants, products and all the intermediates MDA synthesis as well as MDA and MDAH^+^ isomers. Standard enthalpy of formation (Δ_f,298.15K_*H*°(g)) is calculated from G3MP2B3 and CBS-QB3 enthalpies by means of atomization scheme (AS) at 1 atm pressure at 298.15 K. Absolute deviation is given in parenthesis.

Species	Δ_f,298.15K_*H*^0^ (g)	Method	Ref.	*S*_0_(g)	*C*_v_(g)	Ref.
	kJ/mol			J/molK	J/molK	
aniline (A)	86.5 (0.5)	AS(G3MP2B3)	^1^	319.0	96.6	^1^
	96.0 (9.0)	AS(CBS-QB3)	^1^	317.3	97.4	
	87.0 ± 0.88	Burcat	[34]	311.6	104.5	[34]
non-covalent aniline dimer (A_2_)	156.2	AS(G3MP2B3)	311.7	529.6	214.3	^1^
formaldehyde (F)	−111.5 (2.3)	AS(G3MP2B3)	^1^	224.4	26.8	^1^
	−113.3 (4.1)	AS(CBS-QB3)	^1^	224.3	26.8	
	−109.2 ± 0.11	Ruscic ATcT	[28]	218.8	35.4	[35]
4,4′-methylene diphenyl diamine (4,4′-MDA)	171.2	AS(G3MP2B3)	[19]	500.1	221.4	^1^
	191.5	AS(CBS-QB3)		503.6	223.3	
	165.6	additivity rule	[18]	522.7	n.a.	[18]
	172	additivity rule [36,37]	NIST [37,38]	511.6	234.7	[38]
2,4-MDA	159.4	AS(G3MP2B3)	^1^	490.4	220.6	^1^
2′,4-MDA	168	AS(G3MP2B3)	^1^	484.0	219.8	^1^
3,4-MDA	168.3	AS(G3MP2B3)	^1^	499.4	221.3	^1^
N-(p-aminobenzyl)aniline (PABA)	202.4	AS(G3MP2B3)	^1^	496.3	216.5	^1^
	201.3	additivity rule	[18]	514.4	n.a.	[18]
N-hydroxymethylaniline	−71.8	AS(G3MP2B3)	^1^	379.3	129.3	^1^
protonated aniline (AH+)	739.4	AS(G3MP2B3)	^1^	339.9	97.2	^1^
N-methylenebenzeneaminium	828.6	AS(G3MP2B3)	^1^	346.2	107.9	^1^
4-(anilinomethyl)cyclo-hexa-2,5-dien-1-iminium	858.5	AS(G3MP2B3)	^1^	491.2	220.0	^1^
p-aminobenzylaniline (PABAH+)	785.1	AS(G3MP2B3)	^1^	501.2	219.4	^1^
4-methylidenecyclohexa-2,5-diene-1-iminium (MCH+)	802.8	AS(G3MP2B3)	^1^	340.9	113.7	^1^
4,4′-MDAH^+^	814.3	AS(G3MP2B3)	^1^	494.8	225.1	^1^
2,4-MDAH^+^	812.2	AS(G3MP2B3)	^1^	494.7	226.4	^1^
2′,4-MDAH^+^	830.9	AS(G3MP2B3)	^1^	487.1	224.4	^1^
3,4-MDAH^+^	858.6	AS(G3MP2B3)	^1^	534.2	236.5	^1^

^1^ this work.

**Table 3:** Protonation dissociation constants (p*K*_a,aq_) for the protonated intermediates in aqueous solution. The dissociative proton presented in red.

Species		p*K*_a,aq_	p*K*_a,aq_
AH^+^		4.6 ^1^	4.60 [44]
N-methylenebenzeneanilium	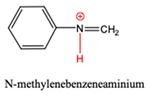	4.2	
PABAH^+^	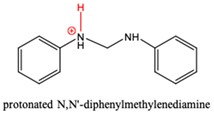	6.7	
MCH^+^	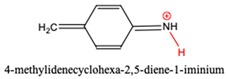	11.4	
AMH^+^	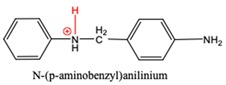	5.1	

^1^ used as reference.

**Table 4:** Thermochemical properties for some side reactions of the formation of 4,4′-MDAH^+^.

Reaction	Aminal (AMH^+^) Formation			IM3H^+^ Formation			OABA^+^ Formation		
	Gas	Aniline	Water	Gas	Aniline	Water	Gas	Aniline	Water
**Δ_r_*E*_0_(kJ/mol)**	−90.7	−75.2	−77.2	32.7	4.9	−1.4	−61.1	−24.9	−26.8
**Δ_r_*H*^0^(kJ/mol)**	−92.2	−76.6	−78.8	29.6	2.7	−4.4	−62.4	−25.8	−28.0
**Δ_r_*G*^0^(kJ/mol)**	-41.1	−26.5	−28.6	42.2	12.0	8.7	−7.7	27.1	26.0

**Table 5:** Energetic description for aniline addition to 4-methylidene-cyclohexa-2,5-diene-1-iminium (MCH^+^) reactions.

Reaction	Δ_r_*E*_0_ (kJ/mol)		Δ^‡^*E*_0_ (kJ/mol)	
	Water	Aniline	Water	Aniline
IM5H^+^ + A → TS6 → IM6H^+^ + A ^1^	−49.2	−61.4	29.2	31.6
A+MCH^+^ → TS6 → 4,4′-MDAH^+^	−62.2	−64.4	17.8	22.5
A+MCH^+^ → TS6* → 2,4 MDAH^+^	−45.1	−49.0	15.5	18.9
A+MCH^+^ → TS6* → 2′,4-MDAH^+^	−52.7	−53.6	−1.9	2.3
A+MCH^+^ → TS6* → 3,4-MDAH^+^	48.4	45.2	54.8	57.4

^1^ IM5H^+^ represents complex of MCH^+^, A and water while IM6H^+^ stands for water complex of 4,4′-MDAH^+^ (see Figure 1).

## Supplementary Materials

The following are available online at https://www.mdpi.com/2073-4360/11/3/398/s1, Figure S1: Transition state structures (obtained at B3LYP/6-31G(d) level of theory) for MDA synthesis in aqueous phase. The G3MP2B3 relative energies are also given, Figure S2: Transition state structures (obtained at B3LYP/6-31G(d) level of theory) for MDA synthesis in aniline. The G3MP2B3 relative energies are also given.
