# Supplementary material for: An Ab Initio Investigation of the 4,4′-Methlylene Diphenyl Diamine (4,4′-MDA) Formation from the Reaction of Aniline with Formaldehyde
Source: Polymers (Basel). 2019 Mar 1;11(3):398. doi: 10.3390/polym11030398 (PMC6473863; doi:10.3390/polym11030398)
Supplement: Supplementary file 1 [file polymers-11-00398-s001.pdf]

polymers

MDPI

Article

An Ab Initio Investigation of the 4,4′-Methlylene Diphenyl Diamine (MDA) Formation from the Reaction of Aniline with Formaldehyde

R. Zsanett Boros[1,2], László Farkas[1], Károly Nehéz[3], Béla Viskolcz[2] and Milán Szőri[2*]

[1] Wanhua-BorsodChem Zrt, Bolyai tér 1., H-3700, Kazincbarcika, Hungary; boros.zsanett0711@gmail.com (R.Zs.B.); laszlo.farkas@borsodchem.hu (L.F.)
[2] Institute of Chemistry, University of Miskolc, Miskolc-Egyetemváros A/2, H-3515, Miskolc, Hungary; bela.viskolcz@uni-miskolc.hu (B.V.); milan.szori@uni-miskolc.hu (M.Sz.)
[3] Department of Information Engineering, University of Miskolc, Miskolc-Egyetemváros Informatics Building, H-3515 Miskolc, Hungary; aitnehez@uni-miskolc.hu (K.N.)
* Correspondence: milan.szori@uni-miskolc.hu; Tel.: +36-46-565-111/1337 (M.Sz.)

Received: 30 January 2019; Accepted: 27 February 2019; Published: 2 March 2019

Supporting Information

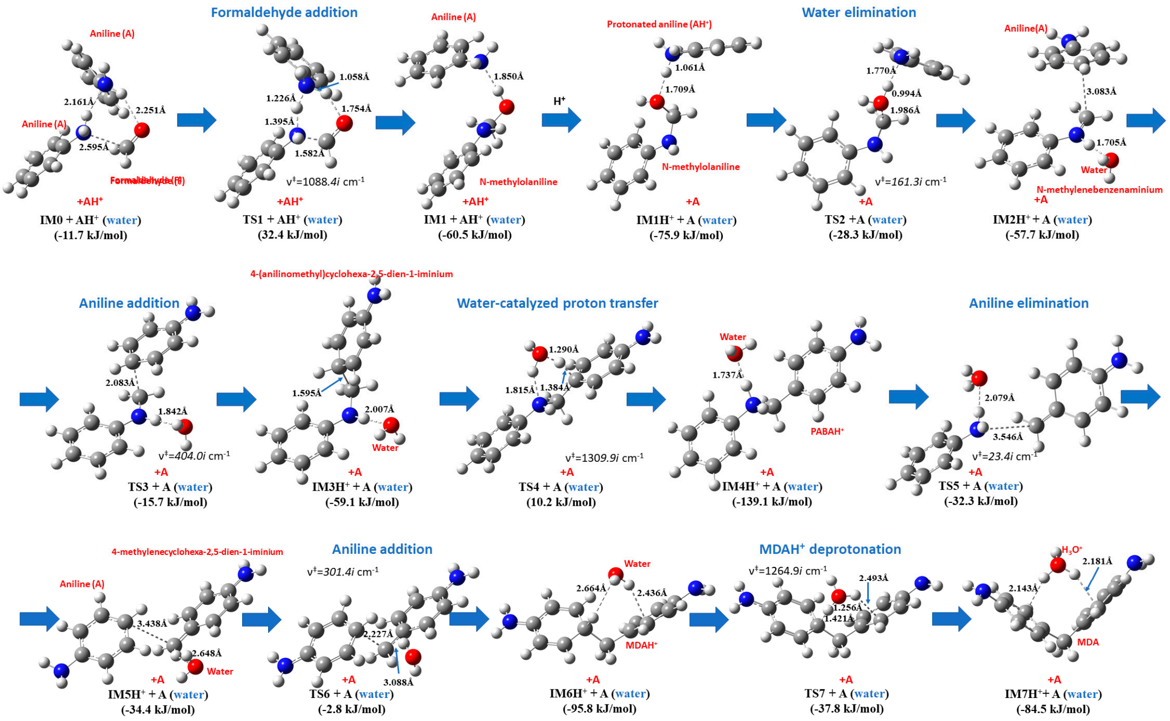

Figure S1. Transition state structures (obtained at B3LYP/6-31G(d) level of theory) for MDA synthesis in aqueous phase. The G3MP2B3 relative energies are also given.

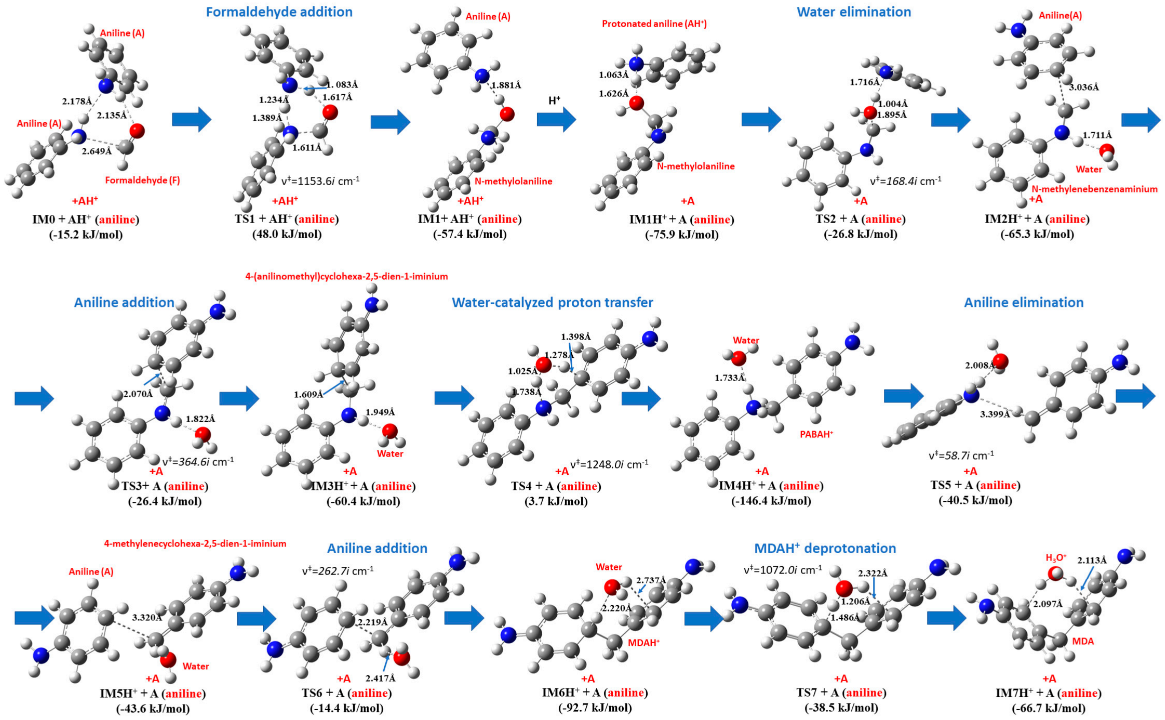

Figure S2. Transition state structures (obtained at B3LYP/6-31G(d) level of theory) for MDA synthesis in aniline. The G3MP2B3 relative energies are also given. .
